# Cell Cycle Regulatory Protein Expression in Multinucleated Giant Cells of Giant Cell Tumor of Bone: do They Proliferate?

**DOI:** 10.3389/pore.2021.643146

**Published:** 2021-05-03

**Authors:** Mate E. Maros, Peter Balla, Tamas Micsik, Zoltan Sapi, Miklos Szendroi, Holger Wenz, Christoph Groden, Ramses G. Forsyth, Piero Picci, Tibor Krenacs

**Affiliations:** ^1^ 1^st^ Department of Pathology and Experimental Cancer Research, Semmelweis University, Budapest, Hungary; ^2^ Department of Biomedical Informatics at the Center for Preventive Medicine and Digital Health, Mannheim, Germany; ^3^ Department of Neuroradiology, Medical Faculty Mannheim, University of Heidelberg, Mannheim, Germany; ^4^ Department of Orthopedics, Semmelweis University, Budapest, Hungary; ^5^ Department of Anatomic Pathology and Experimental Pathology, University Ziekenhuis, Brussels, Belgium; ^6^ Laboratory of Experimental Oncology, Istituto Ortopedico Rizzoli, Bologna, Italy

**Keywords:** giant cell tumor of bone, giant cells, Ki-67, cyclin D1, p53, p21 (CDKN1A) WAF1, cyclin G1

## Abstract

Cells of the monocyte macrophage lineage form multinucleated giant cells (GCs) by fusion, which may express some cell cycle markers. By using a comprehensive marker set, here we looked for potential replication activities in GCs, and investigated whether these have diagnostic or clinical relevance in giant cell tumor of bone (GCTB). GC rich regions of 10 primary and 10 first recurrence GCTB cases were tested using immunohistochemistry in tissue microarrays. The nuclear positivity rate of the general proliferation marker, replication licensing, G1/S-phase, S/G2/M-phase, mitosis promoter, and cyclin dependent kinase (CDK) inhibitor reactions was analyzed in GCs. Concerning Ki67, moderate SP6 reaction was seen in many GC nuclei, while B56 and Mib1 positivity was rare, but the latter could be linked to more aggressive (*p* = 0.012) phenotype. Regular MCM6 reaction, as opposed to uncommon MCM2, suggested an initial DNA unwinding. Early replication course in GCs was also supported by widely detecting CDK4 and cyclin E, for the first time, and confirming cyclin D1 upregulation. However, post-G1-phase markers CDK2, cyclin A, geminin, topoisomerase-2a, aurora kinase A, and phospho-histone H3 were rare or missing. These were likely silenced by upregulated CDK inhibitors p15^INK4b^, p16^INK4a^, p27^KIP1^, p53 through its effector p21^WAF1^ and possibly cyclin G1, consistent with the prevention of DNA replication. In conclusion, the upregulation of known and several novel cell cycle progression markers detected here clearly verify early replication activities in GCs, which are controlled by cell cycle arresting CDK inhibitors at G1 phase, and support the functional maturation of GCs in GCTB.

## Introduction

There are two major ways of forming multinucleate giant cells i.e. acytokinetic cell division and cell fusion [1]. Proliferating neoplastic cells, e.g. Reed-Sternberg cells in Hodgkin’s lymphoma or multinucleated tumor cells in soft tissue, e.g. myxofibro- and osteosarcomas are resulted from incomplete cell division as a result of their cytoskeleton vulnerability e.g. of the contractile ring [2]. On the contrary, inflammatory multinucleated giant cells (GC), such as osteoclast-type giant cells, Langhans-type granuloma giant cells and foreign body giant cells are formed by fusion of cells of the monocyte-macrophage lineage [3].

By testing the expression of cell cycle phase progression associated markers in the mononuclear cells of GCTB, we recently showed that cases with elevated post-G1-phase cell fraction, indicating accelerated cell cycle progression, may predict shorter progression free survival (PFS) [4]. We also recognized that GC nuclei may show diverse proportion of immunoreactions not only for the earlier detected cyclin D and p21^WAF1^ [5, 6], and cell cycle control proteins, but also for some cell cycle licensing and promoter markers, which had not been noticed before, despite GCs are considered to be of reactive, osteoclastic phenotype [7]. Therefore, here we studied the expression of a comprehensive set of cell cycle regulatory proteins to see if GCs in GCTB are still show replicative activity and if it has a clinico-pathological relevance.

**FIGURE 1:**
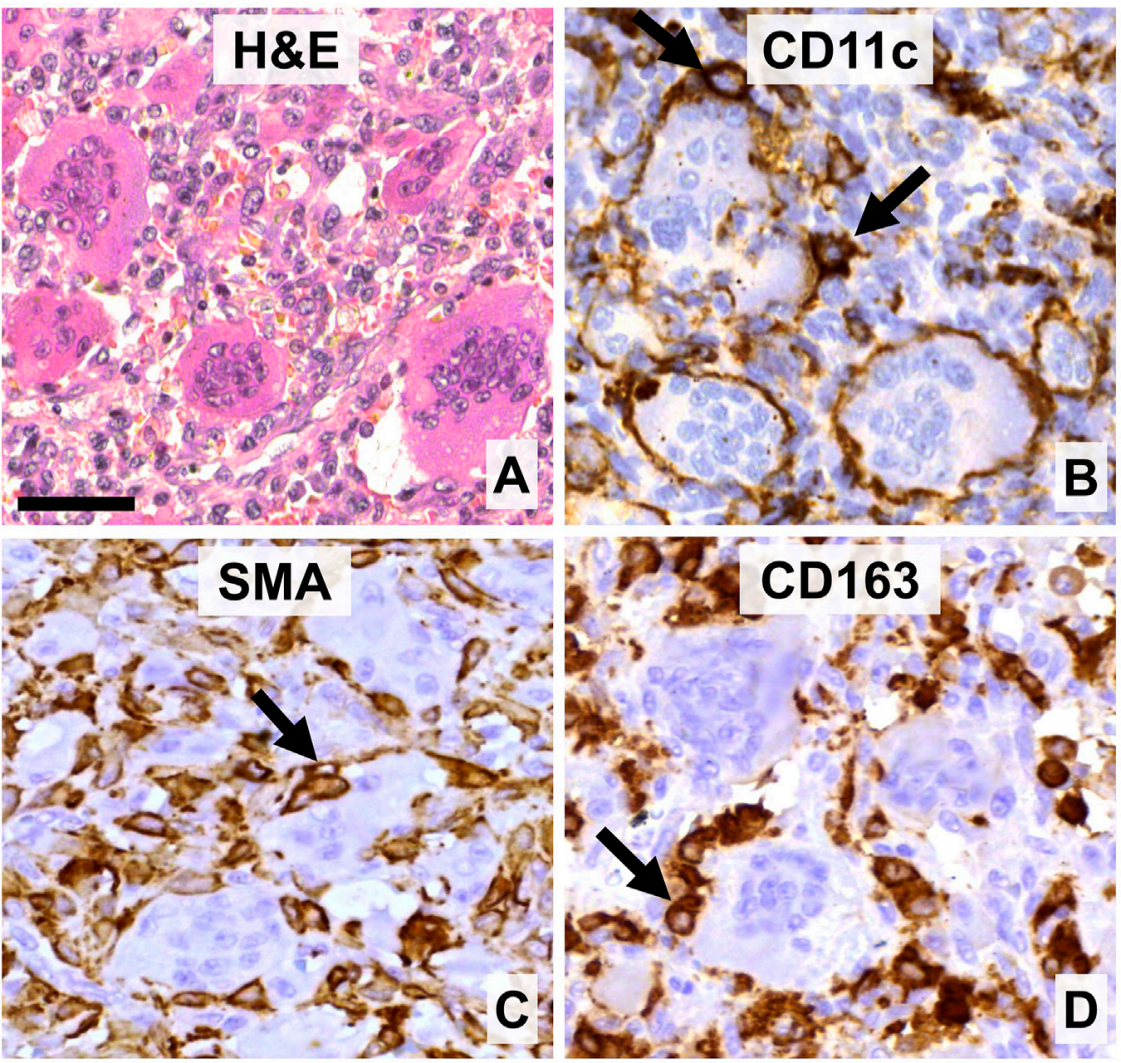
The main cell components of giant cell tumor of bone. **(A)** Multinucleated giant cells (GCs) of different sizes are intermingled with mononuclear and red blood cells (hematoxylin-eosin staining, **(**H&E**)**. **(B)** GCs and their monocytic precursors are positive for CD11c. **(C)** Neoplastic stromal cells can be positive for smooth muscle actin (SMA). **(D)** Histiocytes and monocytic GC precursors but not GCs express CD163 scavenger receptor. Arrows show immunopositive cells intimate association with GCs. DAB (brown) immunoperoxidase reactions **(B–D)**. Scale bar: 50 μm for all images.

GCTB is an osteolytic, locally destructive bone lesion, which, besides GCs, is made up mainly of mononuclear monocytic cells which act as precursors for GCs, and of neoplastic stromal cells (Figure 1). The proliferating, neoplastic stromal cells, generally carrying H3F3A G34W mutation [8], are the major drivers of osteoclastogenesis and pathological bone resorption [9, 10]. They produce canonical (RANKL/M-CSF) and non-canonical (e.g., LIGHT, TNFα, IL-6 or vascular endothelial growth factor–VEGF) growth factors and hypoxia inducible factors 1α and 2α [14], which can either directly or through autocrine activation promote osteoclastogenesis and osteolysis [11, 12]. We have shown earlier that besides the replication activity of neoplastic stromal cells, their elevated epidermal growth factor receptor (EGFR) signaling and deregulated gap junction connexin43 expression and channel functions, can contribute to GCTB progression, mediated by GCs [4, 13, 14].

In the present study, we tested the expression of cell cycle regulatory proteins in GC nuclei, including 3 clones for the general proliferation marker Ki67; the DNA replication licensing factors MCM2 and MCM6; the G1-S phase marker cyclin D1 and its complexing partner CDK4/6; the early (CDK2 and cyclin A) and late (topoisomerase 2–TOP2) post-G1 phase markers; and the G2-M-phase markers (aurora kinase A–AURKA, and phospho-histone-H3–pHH3) [15]. Furthermore, the DNA replication inhibitor geminin, the cyclin dependent kinase inhibitors p15^INK4b^, p16^INK4a^, p21^WAF1^ and p27^KIP1^; as well as the oncosuppressor retinoblastoma and p53 [16], and the unpaired cyclin G1 [17, 18] proteins were also examined. We aimed to profile cell cycle activity in GCs and see if it is different between primary and recurrent GCTB.

## Materials and Methods

### Study Cohort

This study was performed on immunostained 2 mm diameter, 70 sample tissue microarray (TMA) sections of formalin-fixed and paraffin-embedded samples of GCTB cases, which were diagnosed between 1994–2005 at the Institute of Rizzoli, Bologna (IOR), Italy (ethical approval: IOR 13351/5-28-2008 and Semmelweis University: 87/2007) [9,10]. A stratified random sample of 10 primary tumors (P) and 10 first recurrences (1-Rec) were selected from our previously published single-center retrospective study [9]. The clinicopathological characteristics of the selected cohort are presented in Table 1. Briefly, of the 20 patients, 12 progression events were registered (60%) during follow-up. During the study period 12 progression events were registered. Eight-eight patients (40%) were continuously disease free or had local recurrences respectively, 2 (10%) were alive with disease at last follow-up while malignant transformation and stroke both with consecutive fatal outcomes occurred in 1-1 patients (5%).

**TABLE 1:** Clinicopathological features of the study cohort.

Number of patients	20
Number of surgical cases	20
Progression groups	Nr. Enneking’s/Campanacci’s grade
Primary	10 L 3 A 5 Ag 2
1st recurrent cases	10 L 2 A 4 Ag 4
Median age (at case diagnosis)	30.8 years (range: 13.7–75.6 years)
Sex (female, %)	13 (65)
Sex ratio	0.54:1 (m/f) or 1:1.85 (f/m)
Survival	
Median recurrence survival	58.1 months (range:5.5–159.5, IQR: 18.9–79.2)
Number of progression events	12
Localization	Total (%)
Upper limb	5 (25)
Lower limb	13 (65)
Central (Sacrum + Spine or Pelvis)	2 (10)
Treatment types	Total (%)
Curettage	12 (60)
Resection or Amputation or Excision	6 (30)
Radiotherapy	2 (10)

GCTB, giant cell tumor of bone; L, latent; A, active; Ag, aggressive; LQ/UQ, lower/upper quartile

### Immunohistochemistry

Following routine dewaxing, the antigen retrieval of TMA sections was done in an electric pressure cooker (Avair, Bitalon, Pecs, Hungary) using 0.01 M Tris–0.1 M EDTA (TE) at ∼105°C for 30 min. Mouse or rabbit monoclonal primary antibody clones, or rabbit polyclonal immunoglobulins were incubated overnight (16 h) at room temperature. These included anti-Ki67 Mib1 (1:100; Dako, Glostrup, Denmark), B56 (1:100; Histopathology, Pecs, Hungary), SP6 (1:600); anti-MCM2 (CRCT2.1, 1:200) and -MCM6 (1:600, PA5-79649); anti-cyclin D1 (SP4, 1:200), -CDK2 (1:300, 2B6), -CDK4 (1:300, DCS31 + 35), -cyclin E (13A3, 1:20), -cyclin G (11C8, 1:100), -cyclin A (6E6, 1:500), -topoisomerase 2 (Ki-S1, 1:200), -aurora kinase A (1G4, 1:80; Cell Signaling, Danvers, MA, United States), -pHH3Ser10 (K872.3, 1:100); -p53 (DO7, 1:100), -retinoblastoma (1F8, 1:100), -p15^INK4b^ (15P06, 1:200), -p16^INK4a^ (JC8, 1:200), -p21^WAF1^ (SX118, 1:100), and -geminin (EM6, 1:150; Leica-NovoCastra, Newcastle Upon-Tyne, United Kingdom), -p53 (DO7, 1:200, Leica-NovoCastra; and BP53–12, 1:100), and -retinoblastoma (51B7, 1:100) immunoglobulins (IgGs). Except where otherwise indicated, all antibodies were from Thermo-Fisher LabVision (Fremount, CA, United States). Then, the NovoLink polymer peroxidase kit (Leica-NovoCastra) was used as a detection system for 60 min. Immunoreactions were revealed by using DAB Quatro kit (Thermo-Fisher) for 3–5 min under microscopic control and the sections were coverslip mounted after hematoxylin nuclear conterstaining. For double immunofluorescence (C) mouse Ki67 (Mib1, green) and rabbit cyclin D1 (SP4, red) antibodies, were detected simultaneously using Alexa Fluor 488 goat anti-mouse IgG (1:200, green; code: A11001), Alexa Fluor 564 goat anti-rabbit IgG (1:200, red; code: A11035). The immunostained TMA sections were digitalized with the Pannoramic^®^ Scan II System and analyzed using its CaseViewer software (3DHISTECH, Budapest, Hungary).

### Evaluation of the Immunoreactions

After setting up and agreeing on the evaluation criteria with the project leader (TK), systematic assessment was done by an independent assessor (MEM) blinded to all clinical- and other cell cycle marker expression data in three different osteoclast/giant cell rich high-power fields (HPF; 80x) in each case (overall ∼1000 regions of interest) [4]. All nuclear positivity, which was obvious compared to adjacent negative cells showing only hematoxylin staining, was counted. Additional cytoplasmic staining was considered only for p16^INK4a^ on a four-grade Likert scale (0-negative, 1-weak, 2-moderate, 3-strong). The number of GCs (N_GC_), GC nuclei (N_GC_nuclei_) and respective cell cycle marker positive GC nuclei (N_GC_nuclei+_) were recorded and averaged for each case. To robustly estimate N_GC_ and N_GC_nuclei_ in a surgical specimen, their values were averaged over all tested cell cycle markers for each case, respectively. As N_GC_nuclei+_ values are dependent on multiple factors e.g. the position of the TMA core within the specimen, N_GC_ and properties of the CC marker, their absolute values showed substantial variances and were not necessarily comparable across different immunostainings. Therefore, we normalized these values by calculating their ratio for each staining (N_GC_nuclei+_/N_GC_nuclei_) to allow for more stable and direct comparisons across cell cycle markers.

### Statistical Analyses

All analyses were performed with the R statistics program (v.3.6.3, R Core Team 2020, Vienna Austria; RStudio IDE v. 1.2.5033, Boston, MA, United States). Non-normally distributed variables were displayed as median, range and interquartile range (IQR). Categorical variables were reported as proportions. The Jonckheere–Terpstra test was used to investigate the overall difference between Enneking’s/Campanacci’s grading (i.e. latent, active and aggressive), GC count and GC nuclear positivity. We used the nonparametric Wilcoxon-Mann-Whitney *U* test for two samples comparing the mean rank of N_GC_, N_GC_nuclei_, and N_GC_nuclei+_ as well as their ratios between primary and recurrent samples [19]. Uni- and multivariate Cox proportional hazards models of time-to-first-event analyses were performed to explore possible associations between N_GC_nuclei+_ and progression free survival (PFS) [4]. Figures were generated with the ggplot2 library using colorblind-friendly palettes. *p*-values were adjusted for multiple testing to counteract type 1 error inflation using the conservative Bonferroni correction. Adjusted *p*-values (p*) <0.05 were considered significant.

## Results

### General Proliferation Markers in Giant Cells

Based on the potential importance of GC functions in GCTB progression, which may be linked to proliferation, first we counted the number and nuclear density of GCs. Though, neither the overall average GC number (N_GC_; W = 59, *p* = 0.53) nor the average number of GC nuclei (N_GC_nuclei_; W = 49, *p* = 0.97) showed statistical difference between P and 1-Rec GCTB cases, there was a trend of inverse relationship between the radiological grade (latent: L; active: A; aggressive: Ag) of GCTB and the overall average N_GC_ (W_L_vs_Ag_ = 30, p_L_vs_Ag_ = 0.065; W_A_vs_Ag_ = 37, p_A_vs_Ag_ = 0.11) and N_GC_nuclei_ (W_L_vs_A_ = 29, p_L_vs_A_ = 0.093). The distribution of the ratio of cell cycle marker positive GC nuclei in primary and 1-Rec GCTB cases is summarized in Table 2.

**TABLE 2:** Ratios of cell cycle marker positive GC nuclei in primary and first recurrent GCTB.

Type of material	Marker	Ratio of positive GC nuclei				Statistic (W)	*p*	p*_Bonferroni_ (n = 14)	p_adj_
		Median	IQR	min	Max				
P	CDK2	0.031	0.055	0	0.111	72	0.10	0.0036	n.s.
1-Rec	CDK2	0.004	0.012	0	0.078				
P	CDK4	0.325	0.642	0	0.95	28	0.72	0.0036	n.s.
1-Rec	CDK4	0.412	0.193	0.043	0.826				
P	Cyclin A	0	0	0	0	40	0.17	0.0036	n.s.
1-Rec	Cyclin A	0	0	0	0.006				
P	Cyclin D1	0.941	0.13	0.694	0.994	66	0.25	0.0036	n.s.
1-Rec	Cyclin D1	0.874	0.152	0.462	0.981				
P	Cyclin G1	1	0.018	0.868	1	21	0.091	0.0036	n.s.
1-Rec	Cyclin G1	0.956	0.053	0.333	0.967				
P	Geminin	0	0	0	0.005	3	**0.045**	0.0036	n.s
1-Rec	Geminin	0.015	0.017	0	0.061				
P	Ki67 B56	0.027	0.045	0	0.096	22	0.32	0.0036	n.s.
1-Rec	Ki67 B56	0.047	0.017	0.007	0.118				
P	Ki67 Mib1	0	0.012	0	0.048	17	**0.012**	0.0036	n.s.
1-Rec	Ki67 Mib1	0.034	0.039	0	0.172				
P	Ki67 SP6	0.955	0.054	0	1	13	1	0.0036	n.s.
1-Rec	Ki67 SP6	0.75	0.958	0.012	1				
P	MCM2	0.021	0.072	0	0.118	13.5	0.52	0.0036	n.s.
1-Rec	MCM2	0.051	0.158	0	0.225				
P	MCM6	0.5	0.544	0.174	1	20	0.15	0.0036	n.s.
1-Rec	MCM6	0.209	0.265	0.061	0.438				
P	p15^INK4b^	0.773	0.162	0.447	0.967	13	1	0.0036	n.s
1-Rec	p15^INK4b^	0.884	0.942	0	1				
P	p16^INK4a^	0.032	0.045	0	0.233	10	0.69	0.0036	n.s.
1-Rec	p16 ^INK4a^	0.026	0.312	0.009	1				
P	p21^WAF1^	0.818	0.162	0.264	1	32	0.31	0.0036	n.s.
1-Rec	p21^WAF1^	0.902	0.082	0.392	1				

Bold p-values for geminin and Ki67 Mib1 indicate non-adjusted statistical significance.

**FIGURE 2:**
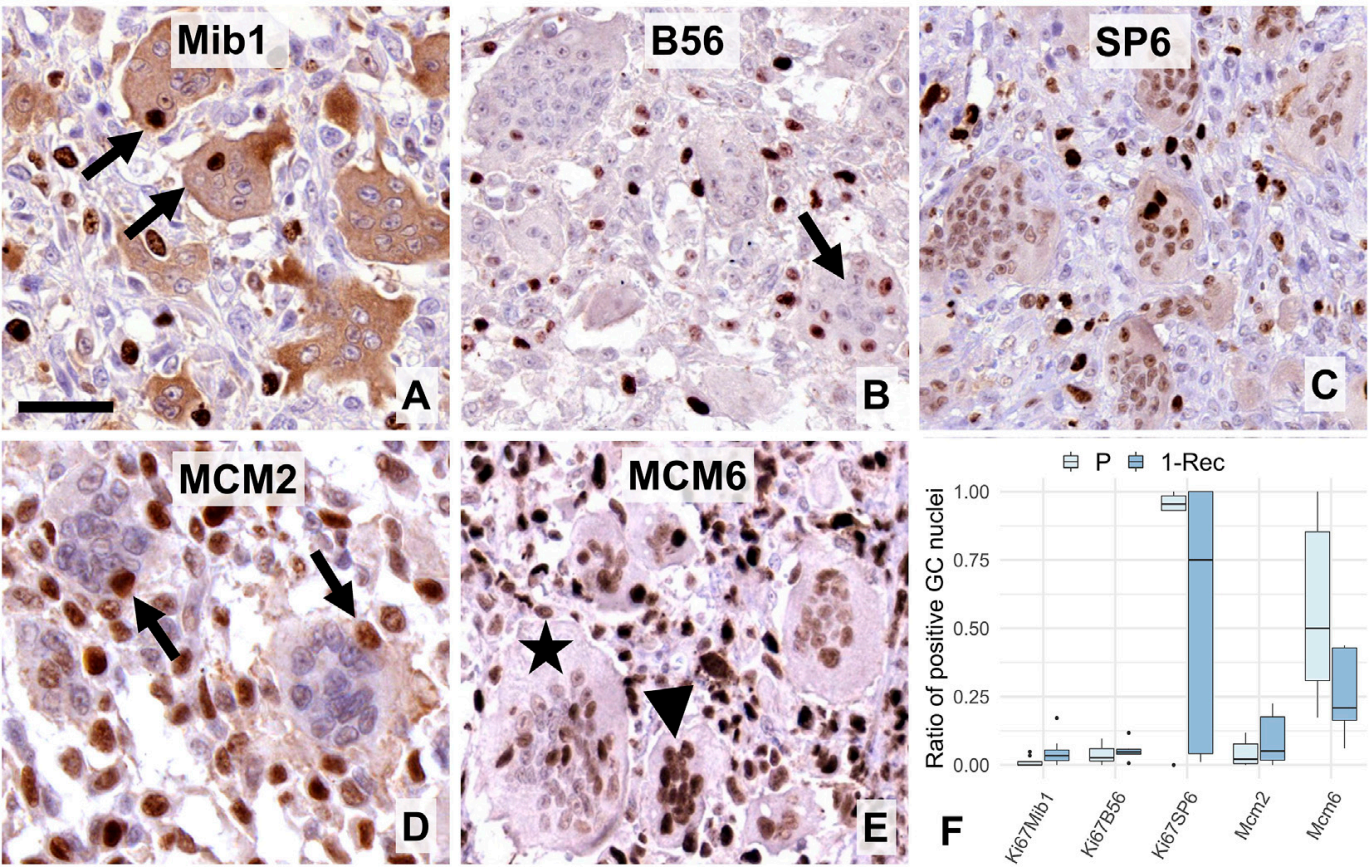
Expression of “general” proliferation markers i.e. Ki67 **(A–C)** and MCM-complex proteins **(D–E)** in multinucleated GCs. Mib1 **(A)** and B56 **(B)** antibodies showed occasional nuclear immunoreactions (arrows), while the SP6 clone resulted in usually weaker, but a widespread Ki67 positivity in GCs. Cytoplasmic Mib1 positivity in GCs was validated by negativity in several mononuclear cells. MCM2 reaction (arrows) was also rare in GCs **(D)**, while that of MCM6 was rather frequent **(E)** and obviously more pronounced in smaller (arrowhead), than larger GCs (asterisk). Scale bar represents 40 μm on A, 50 μm on **(B,C,E)**, and 30 μm on **(D)**. Boxplot of the ratio of immunopositive GC nuclei vs. all GC nuclei **(F)** in primary (P) and first recurrent (1-Rec) GCTB cases.

Of the 3 clones for the general proliferation marker protein Ki67, both mouse monoclonals (Mib1 and B56) showed occasional positive reaction in a few GC nuclei (Figure 2A,B). Interestingly, weak to moderate Mib1 reaction generally appeared also in the cytoplasm of GCs. Unexpectedly, the rabbit monoclonal SP6 reacted in many GC nuclei, however, weaker than in the adjacent proliferating mononuclear cells (Figure 2C). Of note, strong SP6 nuclear staining in GCs was at similar frequency as with Mib1 or B56. Of the replication licensing complex proteins, MCM2 was detected also only occasionally in a few GC nuclei (Figure 2D), as opposed to MCM6 which was seen frequently, but also weaker in the large (>40 nuclei) GCs, than in the small (<10–15 nuclei) ones or in the mononuclear cells (Figure 2E). Though Mib1 positive nuclei in GCs were markedly higher (W = 17, *p* = 0.012) in 1-Rec than in P cases (Figure 2F), it did not reach significance after adjusting for multiple testing (p* = 0.0036). Also, neither B56 (*p* = 0.32) and SP6 (*p* = 1.0) Ki67 clones, nor MCM2 (*p* = 0.52) and MCM6 (*p* = 0.15) positive GC nuclei showed statistically different frequency between P and 1-Rec cases (see also in Table 2).

### G1/S-phase Progression Markers in Giant Cells

**FIGURE 3:**
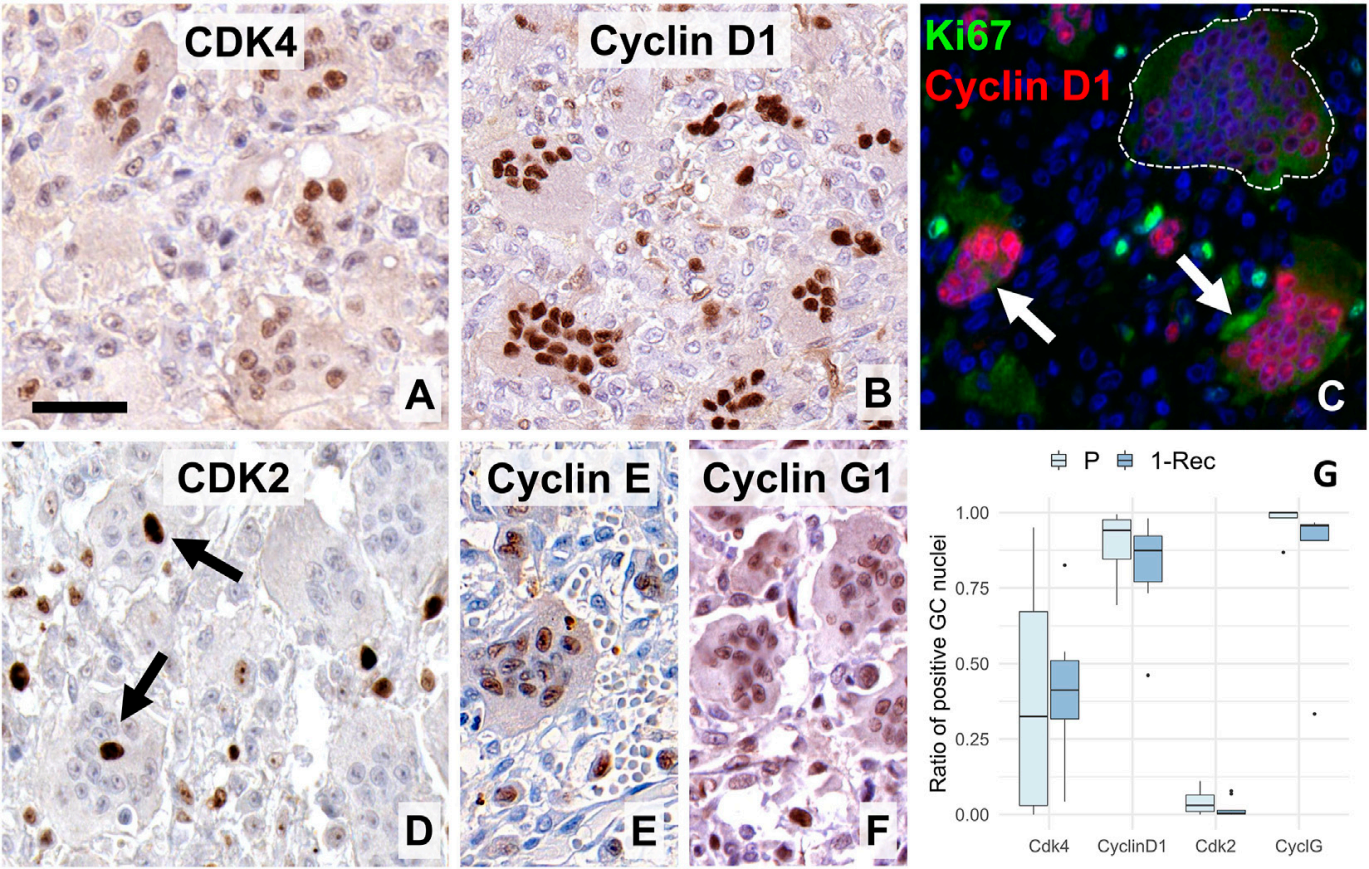
Expression of early G1-S-phase promoting cell cycle markers in giant cells (GC). Many nuclei were positive for the CDK 4/6 **(A)** and almost all were strongly stained for its complexing partner cyclin D1 **(B)**. Double immunofluorescence **(C)** for Ki67 (Mib1, green) and cyclin D1 (SP4, red) showed strong cyclin D1 positivity in smaller GCs (white arrow) but only very faint and missing reaction in a large, aged GC (within white broken line). CDK2 **(D)** was rarely detected in GCs (arrows), as opposed to the widespread expression of cyclin E **(E)** and cyclin G1 **(F)**. Scale bar: 40 μm for **(A,C,D)**; and 50 μm for **(B,E,F)** images. Boxplots of the ratio of immunopositive GC nuclei vs. all GC nuclei **(G)** for some of these markers in primary (P) and first recurrence (1-Rec) GCTB.

Concerning early G1-S phase regulation associated markers, weak to moderate cyclin dependent kinase 4/6 (CDK4/6) reactions were seen in around half of the GC nuclei (Figure 3A), while the vast majority of their nuclei showed moderate to strong reaction with the complexing partner cyclin D1 (Figure 3B). The intensity of the reactions and the rate of positive nuclei for cyclin D1 showed inverse relationship with the size and nuclear density of GCs (Figure 3C). Compared to CDK4/6, much less CDK2 positive nuclei were seen in GCs (Figure 3D). However, its complexing partner cyclin E, though it was not systematically counted, was widely detected in GC nuclei as a moderate reaction (Figure 3E), so as cyclin G1 (Figure 3F). Though average frequency of cyclin G1 in GCs showed a non-significant trend (W = 21. *p* = 0.091) toward P cases, CDK4 (W = 28, *p* = 0.72), cyclin D1 (W = 66, *p* = 0.25) and CDK2 (W = 72, *p* = 0.10) values did not differ statistically between primary and 1-Rec cases (Figure 3G).

### Post G1-phase Markers and Cell Cycle Inhibitors in Giant Cells

**FIGURE 4:**
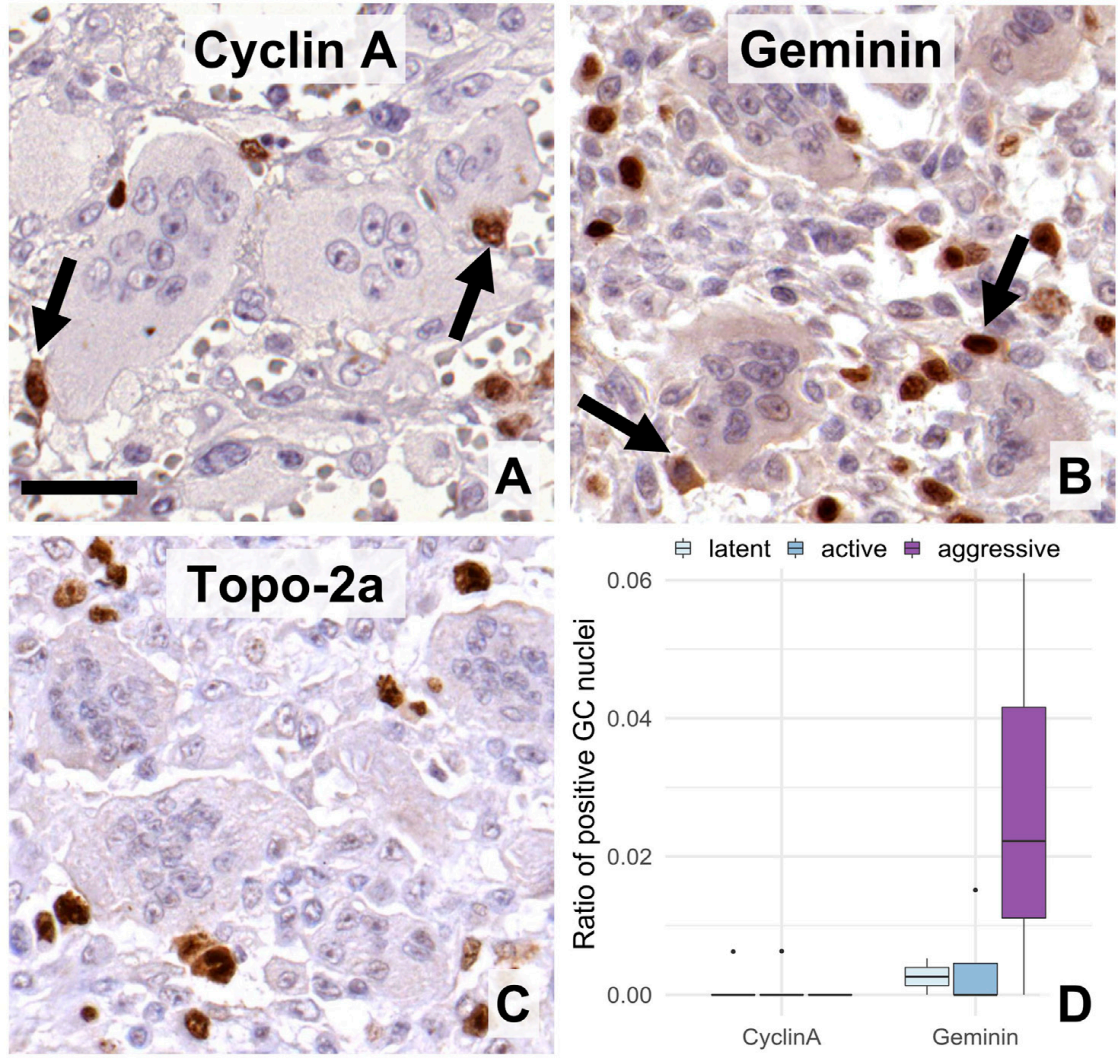
Expression of post-G1 (S-G2-M)-phase cell cycle markers in GC. Cyclin A **(A)**, geminin **(B)**, and topoisomerase-2a **(C)** were practically detected only in the mononuclear cells. Arrows show immunopositive mononuclear cells of close association with multinucleated GCs. Scale bar: 30 μm for all images. Very rare geminin positive cells were somewhat more frequent in agressive grade tumors vs. the other groups (**(D)**, boxlot).

Cyclin A, the S-G2-M transition partner of CDK2, was practically not detected (Figure 4A) in GCs, while the cell cycle repressor geminin (Figure 4B) only at very low frequency, although it appeared more often in 1-Rec cases (W = 3, *p* = 0.045/n.s.). Topoisomerase 2a (Figure 4C), responsible for genome organization in S-phase and chromatid segregation in mitosis, was not detected. Also, as expected from these, both the G2-M phase associated aurora kinase A and pHH3 (the latter primarily labels metaphase cells), were missing from GCs. The latter was also very rare even in the mononuclear cell fraction. Boxplots of quantitative analyses for some of these markers are shown in (Figure 4D).

**FIGURE 5:**
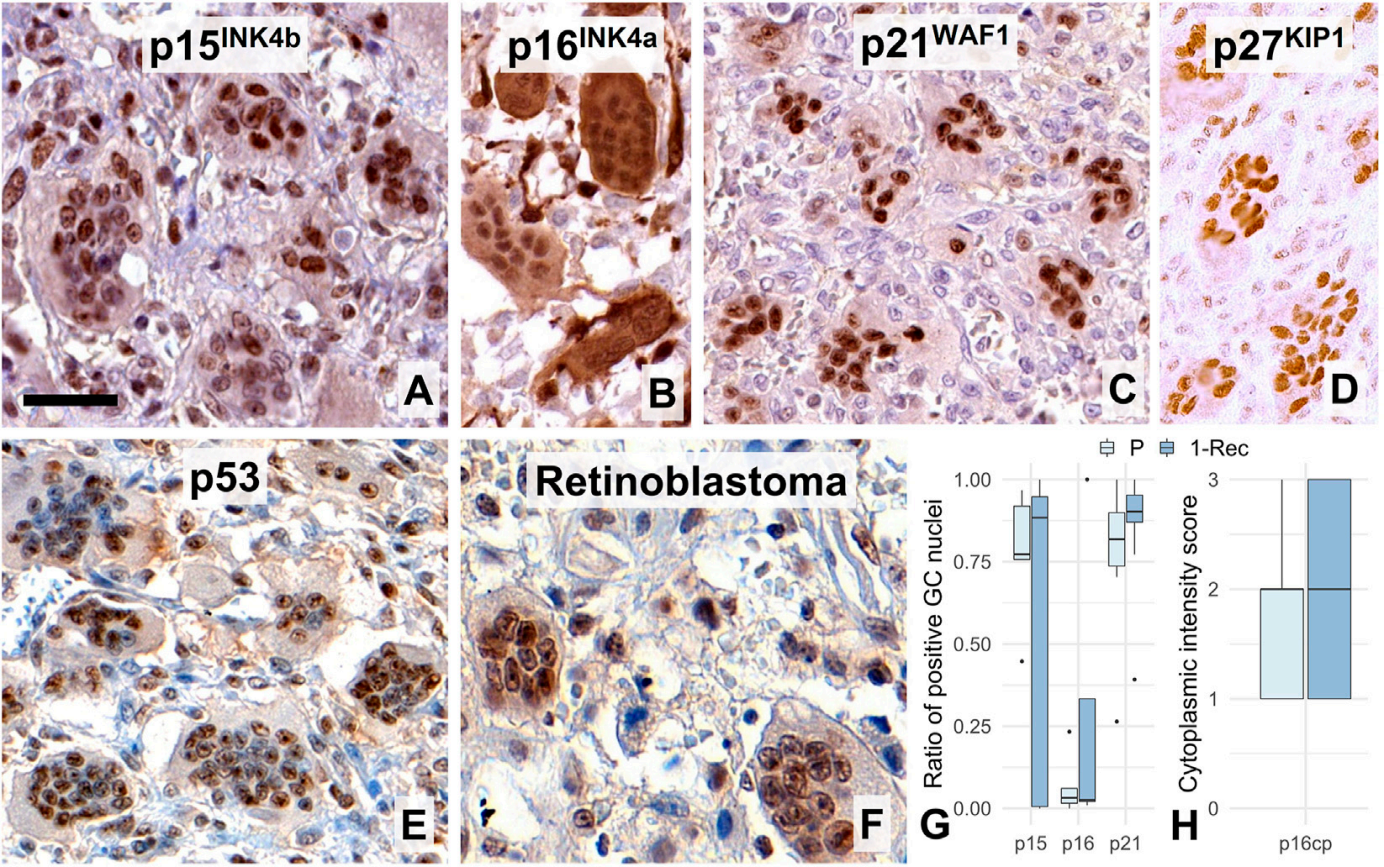
Widespread nuclear immunoreactions for the cell cycle control inhibitor markers p15^INK4b^ **(A)**, p16^INK4a^ (B, where cytoplasmic reaction was frequent), p21^WAF1^, **(C)**, p27^KIP1^ **(D)**, p53 (DO7, **E)**, and retinoblastoma **(F)** proteins in multinucleated giant cell nuclei. Scale bar represents 40 μm on **(A)**, 50 μm on **(B–E)**, and 30 μm on **(F)**. Boxplots of the ratio of immunopositive GC nuclei vs. all GC nuclei for some of these markers **(G)**; and for the cytoplasmic p16^INK4a^ reaction **(H)** in primary (P) and first recurrence (1-Rec) GCTBs.

In line with these, all CDK inhibitors tested including p15^INK4b^, p16^INK4a^, p21^WAF1^ and p27^KIP1^ were detected widely in GC nuclei (Figures 5A–D). p16^INK4a^ showed the least nuclear positivity but showed widespread cytoplasmic reaction, while p21^WAF1^ was strongly detected practically in most GC nuclei. Fitting into this pattern, the majority of GC nuclei were immunopositive over a wide range of intensities when using either the DO7 (Figure 5E) or BP53–12 antibody clones (not shown) specific for the p53; or when retinoblastoma antibody was used (Figure 5F). However, none of the systematically analyzed CDK inhibitors including p15^INK4b^ (W = 13, *p* = 1.0), p16^INK4a^ (W = 10, *p* = 0.69) and p21^WAF1^ (W = 32, *p* = 0.31) showed differential expression between primary and 1-Rec samples (Figures 5G,H).

### Explorative Survival Analyses

Explorative univariate Cox proportional hazards analyses revealed that the increased average number of Ki67 Mib1 positive GC nuclei (HR = 1.1, 95% CI: 1–1.2, p_non-adj_ = 0.041) was significantly associated with shorter PFS while no other marker showed relevant associations with PFS.

## Discussion

**FIGURE 6:**
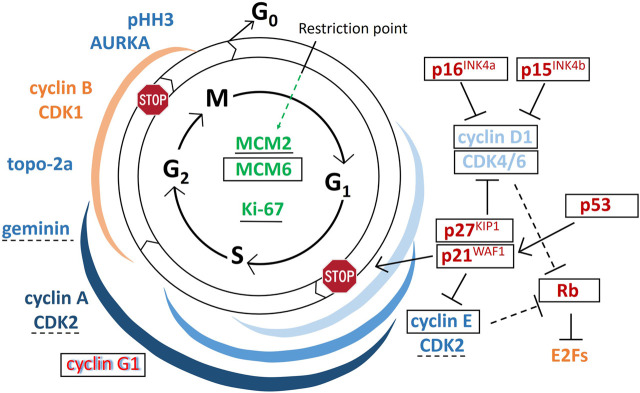
Draft on the emergence and potential role of regulatory proteins during the cell cycle. Arrows indicate activating functions while “T” signs show inhibitory functions. Markers in green can be detected throughout the cycle, those in blue support, while those in red inhibit cell cycle progression; red letters with blue shadow at cyclin G1 indicate binary functions; and the markers in yellow are important but not tested in this study. In GCTB giant cells, the markers that are framed in black were widely detected, those underlined with continuous line were occasionally found, those underlined using broken lines are found very rarely, while those which are not labeled either of these ways, were practically not detected within GCs. Colored ribbons show the rough expression duration of the matching-colored cyclin-cyclin dependent kinase complexes.

In GCTB, GCs need continuous supply of precursors to fuse, progressively form and resorb bone. They are under the influence of growth factors including receptor activator for NFκB ligand (RANKL) and macrophage colony stimulating factor (M-CSF) and their substitutes e.g. interleukins 6, 11 and 8, and TNFα; as well as VEGF, placental growth factor (PlGF), hepatocyte growth factor (HGF) and FLt-3 ligand, respectively, some of which can induce proliferation [11, 20, 21]. Earlier studies by others showed the widespread expression of cyclin D1 [5, 6, 22–24] cyclin D3 [5] and p21^WAF1^ [6], less p16 and scarce Ki67 [22] in GCs. Still, these data were insufficient to declare replication activity in GCs. In our present work, in addition to confirming previous observations, we revealed further cell cycle promoters, which can verify [23] an early cell cycle course in GCs. However, the effect of the generally detected cell cycle licensing MCM6, and the cell cycle promoters CDK4 and cyclin E, are likely neutralized in GCs by the upregulation CDK inhibitors p15^INK4b^, p16^INK4a^, p27^KIP1^ and the p53 induced p21^WAF1^, consistent with a cell cycle arrest at the late G1 phase. The potential emergence and links among the markers of this study during the cell cycle are drafted in Figure 6.

The general proliferation marker Ki67, which has been involved in heterochromatin organization, the inhibition of p21-mediated G1/S-phase checkpoint activation and in the formation of mitotic perichromosomal protein sheet, can be detected throughout the replication cycle [25]. So far, only one study reported a weak Ki67 Mib1 staining in <5% of GCs in only 3 out of 29 (10%) GCTB samples. Of the 3 Ki67 clones we investigated in GCs, both Mib1 and B56 showed a low ratio of nuclear positivity (<10%) while SP6 demonstrated a wide range with >75% median. The specificity of the moderate intensity SP6 reaction in GCs was validated by the clear negativity of some adjacent mononuclear cells. Though Ki67 expression peaks at G2/M-phases, the protein is only gradually eliminated from newly divided cells, therefore, its amount may also reflect the time the cell spent in quiescence [26]. Accordingly, the markedly higher ratio of positive GC nuclei in 1-Rec vs. primary cases, and its association with shorter PFS in our explorative survival analysis may also indicate accelerated dynamics of GC formation in the advanced cases. The cytoplasmic Mib1 staining in GCs shown also by others [27] is likely to be related to its metabolic elimination by the ubiquitin proteasome system [28]. The differential occurrence of the Ki67 clones can be partly related to the slower degradation of the epitope region recognized by SP6 compared to the others. However, further signs in this study suggested that this may also be linked with an initial replication activity.

Members of the MCM2-7 helicase complex, which can also be detected all over the cycle, unwind double-stranded DNA and allow the controlled licensing of DNA for duplication [16]. However, while MCM6 complexed with MCM4 and MCM7 is involved in relaxing DNA to single strands, the MCM2 subunit (MCM3, and MCM5 too), has potential inhibitory role on this function [29]. Our widespread detection of MCM6 with moderate intensity but only occasional occurrence of MCM2 in GC nuclei, may reflect the initiation of DNA unwinding in GCs.

Cyclin-dependent serine/threonine kinases complex with their regulatory subunit cyclins to phosphorylate retinoblastoma and promote the transition of different phases during cell cycle progression [30]. Here, we revealed the widespread emergence not only of the previously detected cyclin D1 [5, 6], but also its partner CDK4 in the earliest G1-S phase promoter complex. This complex not only can inactivate retinoblastoma but also support the activation of the next G1-S-phase promoter cyclin E-CDK2 complex through reducing mitochondrial metabolism to prevent cyclin E degradation [31]. In agreement with this, we regularly detected cyclin E in GCs, however, only very rarely found its complexing partner CDK2 in GC nuclei, indicating a late G1-phase arrested cell cycle. At the same time, cyclin D1-CDK4/6 complex can also support cell growth e.g. through mTORC1 activation [32], and high cyclin D1 levels can assist in metabolic substrate utilization toward mitochondrial amino acid production [31], which propose a role for cyclin D1 also in GC growth and differentiation. Furthermore, cyclin D1 overexpression in GCs may also be required for multinucleation [5], as it enhanced the number of nuclei e.g. in cardiomyocytes [33] and was detected primarily in giant trophoblasts rather than in diploid ones [34].

In agreement with earlier studies, in GCs we also revealed the widespread expression of p21^WAF1^, the universal CDK inhibitor [35]. However, despite p21^WAF1^ is considered to block cyclin D1-CDK4/6 complex and CDK2 activities, paradoxically it may also be important for CDK4/6 complex assembly [36] and for the nuclear export of cyclin D1 [37, 38]. These might explain our frequent detection of CDK4 but not the CDK2, and the high levels of the CDK4 partner cyclin D1 in GC nuclei. P21^WAF1^ is an important effector of the cell cycle control functions of p53 [39]. Before us, p53 expression had been noticed only incidentally in one publication [40]. Here, we detected p53 in the majority of GC nuclei using 2 different antibody clones (DO7 and BP53–12 respectively) to give, for the first time, clear evidence that GCs upregulate this important “guardian” of the genome. Cytoskeletal stress, which may occur during multinucleation in GCs, can induce p53 upregulation and activate p21^WAF1^ for blocking S-phase entry at G1-checkpoint [39]. The p53-dependent G1 arrest of multinucleated tetraploid cells has been previously described [41] and p53 activity with the contribution of p21^WAF1^ can also drive this process even further to result in cellular senescence [42]. We also noticed the general upregulation of p27^KIP1^ in GCs, which in normal cells is primarily linked to controlling the cell cycle through inhibiting both cyclin E-CDK2 and cyclin D1-CDK4/6 activities [43]. Both p21^WAF1^ and p27^KIP1^ play essential roles also in GC functions as shown by the osteopetrotic phenotype in mice with deleted genes encoding these proteins [44]. In GCTB, arresting of the potential cell cycle activities and inducing senescence are required for GC maturation involving the production of key proteases cathepsin K and MMP-9 for pathological bone resorption [45].

As a p53 target, cyclin G1 has been involved in both supporting cell cycle arrest and in driving the S-G2-M-transition [17]. As a supposed oncogene cyclin G1 may activate the MDM2 oncoprotein by recruiting Ser/Thr protein phosphatase 2A (PP2A), which dephosphorylates MDM2 to inhibit and degrade p53 [17, 18, 46]. We detected cyclin G1 in most GC nuclei with a non-significant trend toward higher values in primary vs. 1-Rec GCTB cases. This may rather support its role in cell cycle arrest than as a promoter of replication in GCs which are reactive, non-malignant cells. However, it is also possible, that cyclin G1 upregulation is a rebound effect to control p53 overexpression and prevent apoptosis induction [17, 18, 46]. The permanently elevated cyclin D1 levels in functionally active GCs, but its disappearance from oversized (>40 nuclei), aged GCs may also be consistent with the protective function of cyclin D1 [17, 46].

Besides p21^WAF1^ and p27^KIP1^, the upregulation of other CDK inhibitors, particularly targeting CDK4 and CDK6 proteins including p15^INK4b^ and p16^INK4a^, further support the cell cycle related activity in GCs [47]. Both p15^INK4b^ and p16^INK4a^ are also likely to contribute to G1-phase cell cycle arrest [48, 49], while p16^INK4a^ may also be linked to cellular senescence induction supporting either the full functional differentiation or the aged-cell decay in GCs [50]. Cell cycle arrest by CDK inhibitors was also reflected by retinoblastoma upregulation in GCs. In line with these findings, practically none of the S-G2-M phase markers including cyclin A, the later complexing partner of CDK2, the cell cycle repressor geminin, the G2-M phase transition associated cyclin B, or the M-phase related AURKA and pHH3 were detected within GCs [16]. This pattern is consistent with the full functional maturation of GCs. The S-G2 phase marker positive mononuclear cells which had no discernible cytoplasmic boarders with GCs, were likely to be monocytes close to or within the fusion process (Figures 4A,B). In support of this, a subpopulation of CD14+/CD33 + monocytes was shown to proliferate in response to M-CSF and form pre-osteoclasts when primed with RANKL [51]. Then pre-osteoclasts loose CD33 expression and fuse with more monocytic cells [52] and the proliferating monocytes form significantly more GCs than the rest of the monocytic pool [51].

Despite silenced, the early signs of replication proved here, may reflect the generation dynamics and age-related activity of GCs. *In vitro* data confirm an inverse correlation between GC size and resorption activity at mild acidic conditions [53]. This is in line with data showing elevated recurrence potential in GCTB cases, which dominantly contain GCs of <15 nuclei compared to those carrying larger ones [18]. In the present study, the average size and nuclear density of GCs also revealed an inverse trend with the clinicoradiological grade of GCTB. Furthermore, the cyclin D1 and MCM6 reactions in GCs were obviously stronger and more frequent in small sized (∼<15 nuclei) than in larger GCs, particularly when nuclear numbers were ∼>40. These are also in agreement with our earlier finding of the significantly lower average size of GCs in recurrent GCTB cases where the growth related EGFR protein level was elevated in the mononuclear stromal cells, the major drivers of GC formation and activity [13]. All these support the view that smaller sized GCs are the younger, dynamically forming populations, which may show more signs of early replication than the aged, functionally less active oversized GCs (∼>40 nuclei).

Dissecting the replication cycle into major cells fractions through the immunohistochemical detection of nuclear proteins which regulate or control different phases of replication, may allow accurate assessment of cell proliferation dynamics *in situ*. Using a marker set, selected carefully from the list we described here, can serve cell cycle analysis for diagnostic, prognostic and predictive purposes in any pathological process particularly in cancer.

## Conclusion

Though multinucleated GCs in GCTB are thought to be of reactive phenotype formed by fusion of cells of the monocyte macrophage lineage, they had been occasionally shown to express cell proliferation related markers. By using a comprehensive marker set, here we revealed, for the first time in GCs, the general upregulation of cell cycle promoting markers MCM6, CDK4 and cyclin E, indicating primary DNA unwinding and G1-S-phase promoting activities. We also confirmed the earlier published widespread expression of cyclin D1, which all, unequivocally demonstrated an early replication activity in GCs. This, however, was silenced by the widespread expression of CDK inhibitors p15^INK4b^, p16^INK4a^, p27^KIP1^ and p53 induced p21^WAF1^ resulting in cell cycle arrest at the G1 checkpoint, confirmed by the missing production of post-G1-phase faction markers. The complex interplay among these elements under the influence of external growth and differentiation factors is required for the functional maturation and bone resorbing activity of GCs in GCTB.

## Data Availability

The original contributions presented in the study are included in the article/Supplementary Material, further inquiries can be directed to the corresponding authors.
